# Incidentally detected right partially fused, malrotated, supernumerary kidney

**DOI:** 10.4102/sajr.v29i1.3054

**Published:** 2025-02-17

**Authors:** Nishanth Raavani Kumaraswamy, Sushmita Balol, Vittal Manohar, Yashwanth Naik, Shubha Tavarakere Shamasundara

**Affiliations:** 1 Department of Radiodiagnosis, Mysore Medical College and Research Institute, Mysore, India

**Keywords:** supernumerary kidney, congenital anomalies, renal anomalies, genitourinary system, malrotated kidney

## Abstract

Supernumerary kidney (SK) is an uncommon anomaly of the urinary tract, usually occurring on the left side. A rare case of a partially-fused, SK on the right side is presented. The diagnosis is made through the identification of a distinct pelvicalyceal system along with its supplying renal artery and vein, utilising ultrasound, CT, or MRI.

**Contribution:**

Correct diagnosis of partially-fused, supernumerary kidneys based on imaging is crucial to avoid unnecessary procedures, so that asymptomatic cases are managed conservatively.

## Introduction

Congenital anomalies of the upper urinary tract are among the most common anomalies, accounting for ~50% of all congenital anomalies. They can be associated with anomalies of the genital tract because of their common embryological origin.^1^ Supernumerary kidney (SK) is a rare congenital anomaly that shows the presence of an extra kidney, usually on the left side. It may be completely separated from the ipsilateral kidney or partially fused.^2^ Differential diagnoses for SK include conditions such as a duplicated collecting system, crossed fused renal ectopia, and renal tumours or masses mimicking accessory renal tissue. Each of these conditions has distinguishing imaging features that help differentiate them from true SKs.

## Ethical considerations

This case report was approved by Institutional Ethics Committee, Mysore Medical College and Research Institute and associated hospitals, Mysore. Ethical committee clearance was obtained on 15 October 2024. The report followed all ethical standards for research. Informed consent was obtained from the patient and all images have been anonymised.

## Patient presentation

A 32-year-old male patient complained of abdominal pain for 1 month. There was no fever, vomiting, bowel or bladder disturbance. The patient had no comorbidities or prior surgeries. General physical examination, abdominal examination and baseline blood parameters were within normal limits.

**FIGURE 1:**
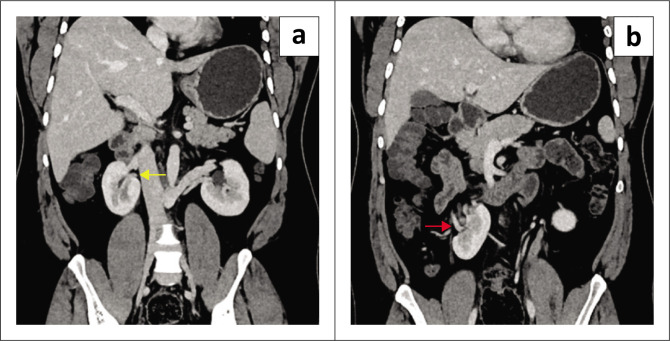
Venous phase contrast-enhanced CT. (a) Coronal reconstruction demonstrating the pelvicalyceal system of the cranially located right kidney facing medially (yellow arrow). (b) Coronal reconstruction with the pelvicalyceal system of the caudally located right kidney facing laterally (red arrow).

**FIGURE 2:**
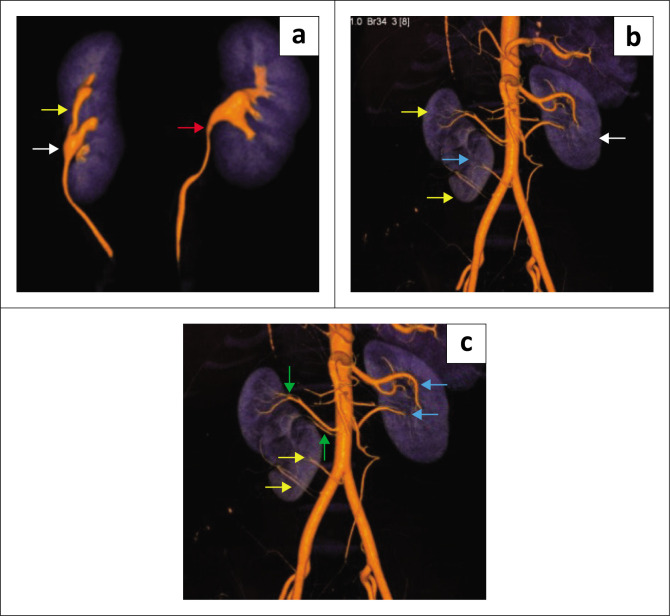
(a) Contrast-enhanced CT of the excretory phase-3D volume rendering (oblique view) of both pelvicalyceal systems and ureters indicating a normal left pelvicalyceal system and ureter (red arrow). On the right, the ureter from the cranially placed kidney (yellow arrow) passes anteriorly and joins the pelvis of the caudally placed kidney at the pelviureteric junction (white arrow) to form a single ureter. (b) 3D volume rendering (oblique view) showing a normal left kidney (white arrow) and two right kidneys (yellow arrows) partially fused by a parenchymal bridge between them (blue arrow). (c) 3D volume rendering (oblique view) showing branches from the abdominal aorta supplying the cranially placed right kidney (green arrows) and branches from right common iliac artery (yellow arrows) supplying the caudally placed right kidney. Two left renal arteries (blue arrows) were found to supply the left kidney.

The patient was subjected to multiphasic contrast-enhanced CT of the abdomen and pelvis. Two kidneys were found on the right side. The upper right kidney measured 6.7 cm × 4 cm, with the hilum facing anteromedially (Figure 1a). The lower right kidney measured 6.2 cm × 4.1 cm with the hilum facing anterolaterally (Figure 1b). Both the right kidneys were partially fused with each other by a small parenchymal bridge at the level of the L2–L3 intervertebral disc. Both right kidneys demonstrated good concentration and prompt excretion of contrast. The two right pelvicalyceal systems joined at the level of the L3 vertebral body to form a single, normal-appearing distal ureter (Figure 2).

**FIGURE 3:**
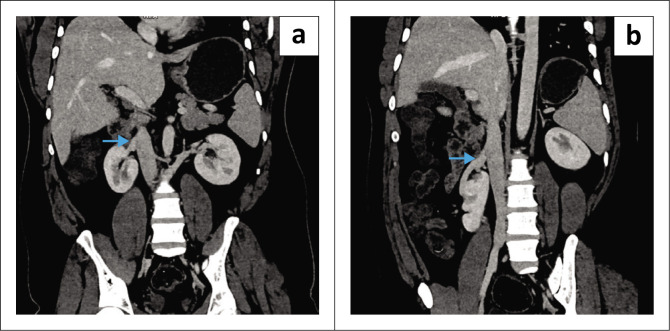
(a) Venous phase contrast-enhanced CT coronal reconstruction revealing the renal vein (blue arrow) draining the cranially placed right kidney joining the inferior vena cava. (b) Coronal reconstruction showing a second renal vein (blue arrow) draining the caudally placed right kidney to the inferior vena cava.

Two renal arteries supplied the cranially and caudally placed right kidneys arising from the abdominal aorta and right common iliac artery, respectively (Figure 2). Two separate right renal veins were found draining into the inferior vena cava, one from each right kidney (Figure 3).

The left kidney appeared normal in size, shape and position with good concentration and prompt excretion of contrast. No pelvicalyceal or ureteric dilatation was observed bilaterally. No other anomalies were seen in the abdomen.

A diagnosis of a right partially-fused, malrotated, SK was made. There were no renal complications such as calculi, pyelonephritis or hydroureteronephrosis, and no pathology was detected in abdomen and pelvis. The patient was therefore managed conservatively. Follow-up with ultrasonography is recommended for early detection of any renal complications.

## Discussion

A SK denotes the presence of one or more extra kidneys. It is an uncommon congenital anomaly (< 100 cases reported) characterised by an extra kidney with a distinct vascular supply and collecting system.^1,3^ Normally the intermediate mesoderm gives rise to nephrogenic cords on both sides, which later forms the pronephros (in the cervical region), mesonephros (in the thoracolumbar region) and metanephros (in the sacral region). The pronephros regresses. The mesonephros drains into the mesonephric duct, which gives rise to the ureteric bud. The metanephros forms in the sacral region in the 5th week of embryogenesis and develops into the permanent kidney. The metanephric blastema interacts with the ureteric bud and forms the nephrons (glomeruli, proximal and distal convoluted tubules). The ureteric bud branches and forms the collecting system. The kidneys are drawn apart, ascend, and rotate to their normal position in the paralumbar regions. During ascent, the kidneys receive blood supply, initially from the common iliac arteries, later from successively higher branches and finally from the abdominal aorta, while the caudal branches degenerate. Once the kidneys reach their final position, one branch on each side becomes the main renal artery and the rest of the branches usually regress^1^. The kidneys rotate during ascent and the hila are directed anteromedially. Congenital anomalies of the upper urinary tract can be classified as anomalies of renal form, renal position, renal number and renal collecting system.^1^

Supernumerary kidney develops due to abnormal division of the nephrogenic cord during the 5th to 7th week of gestation, with each branch of the ureteric bud penetrating independently in the metanephric blastema resulting in two kidneys.^1,4^ An alternate theory is two separate ureteric buds penetrating the metanephric blastema and dividing it into two.^1^

Supernumerary kidney is more common on the left side than on the right.^5,6^ It can be isolated from the ipsilateral kidney or partially-fused with it. Supernumerary kidney may be associated with other congenital anomalies of the genitourinary tract such as horseshoe kidney, cloacal anomalies, ambiguous genitalia, urethral atresia, vaginal atresia, duplication of the urethra and penis, aortic coarctation, imperforate anus, meningomyelocele, among others. Rarely, there could be bilateral fused SKs.^1,6,7,8^ Supernumerary kidney is usually asymptomatic but can present with abdominal pain, mass, haematuria, fever or hypertension. Associated complications include urinary complications such as urinary incontinence, recurrent urinary tract infections, pyelonephritis, pyonephrosis, hydronephrosis, calculi, and rarely neoplasms such as Wilms tumour, clear cell carcinoma and urothelial carcinoma.^1,3,6^ Functional abnormalities may lead to impaired drainage, secondary hypertension, or ectopic ureteral drainage causing incontinence.^9^

The first imaging modality for renal anomalies is ultrasonography due to its non-invasiveness, lack of radiation and cost-effectiveness. The use of intravenous urography has decreased due to alternative imaging modalities. Voiding cystourethrography is used for urethral abnormalities and vesicoureteric reflux.

Imaging with CT and MRI is preferred as assessment of renal anomalies and complications can be made^5^ and CT or MR angiography can help define the vasculature while CT or MR urography delineates the renal collecting systems. Due to a lack of radiation exposure, MR urography may be preferred in children. Nuclear scintigraphy is an alternative imaging modality to evaluate renal function, particularly useful in cases of obstruction or suspected non-functioning kidneys.

Cross-sectional imaging is the mainstay of diagnosis of SK. The SK is usually smaller than the native kidney with its own arterial supply from the aorta or common iliac artery, separate venous drainage into the inferior vena cava, a distinct pelvicalyceal system and encapsulated parenchyma. It may be completely separated from the ipsilateral native kidney or partially-fused with it by fibrous and parenchymal tissue.^1^ It may have partially or completely duplicated ureters. The main differential diagnosis is a duplex kidney in which the pelvicalyceal system is duplicated with partially or completely duplicated ureters but without separate arterial supply and venous drainage.^6,10^ Crossed fused ectopia will have an absent kidney on one side, with each ureter draining to either side of the urinary bladder.^1^

Correct diagnosis with imaging is required as misdiagnosis such as tumour may lead to unnecessary procedures.^11^ Imaging is needed to study the pelvicalyceal system and vascular anatomy, renal functional status, associated congenital anomalies and complications, and to determine management and surgical planning. Asymptomatic cases can be managed conservatively. Symptomatic cases may require surgical treatment and a non-functioning kidney may be treated with nephrectomy.^3,5^ In children, follow-up with ultrasonography is recommended to detect complications.^4^

## Conclusion

Supernumerary kidney is an uncommon congenital anomaly, usually on the left side, but can occur on right. The two kidneys on the same side may be separate from each other, or partially-fused. They are usually asymptomatic, but can result in symptoms and complications. Imaging is indicated to correctly diagnose the condition, to study the anatomy and functional status, and to identify associated anomalies and complications. Asymptomatic cases are managed conservatively, while symptomatic cases may require surgery. Follow-up with ultrasonography can be performed to detect complications early.

## References

[1] Houat AP, Guimarães CT, Takahashi MS, et al. Congenital anomalies of the upper urinary tract: A comprehensive review. RadioGraphics. 2021;41(2):462–486. 10.1148/rg.2021200078 33513074

[2] Suresh J, Gnanasekaran N, Dev B. Fused supernumerary kidney. Radiol Case Rep. 2011;6(4):552. 10.2484/rcr.v6i4.552 27307933 PMC4899937

[3] Berhe T, Hassen SM, Gebrehiwot FG, et al. Fused supernumerary kidney with single pelvis and ureter; presenting with stones: A case report and literature review. Res Rep Urol. 2021;13:853–857. 10.2147/rru.s347328 34993158 PMC8711840

[4] Krakhotkin DV, Chernylovskyi VA, Pikhovkin DN, Ermolaev AN, Bugaev RA. Left supernumerary kidney: A rare case presentation. Radiol Case Rep. 2021;16(3):615–617. 10.1016/j.radcr.2020.12.064 33425084 PMC7785881

[5] Yener S, İlçe Z. A rare case: Supernumerary right kidney in a child. Urol Case Rep. 2021;38:101677. 10.1016/j.eucr.2021.101677 33912397 PMC8066408

[6] Gao X, Xing Q, Luo X, et al. Right supernumerary kidney with urothelial carcinoma. Medicine. 2020;99(38):e22329. 10.1097/md.0000000000022329 32957401 PMC7505325

[7] Tefera AT, Gebreselassie KH, Issack FH, et al. Bilaterally fused supernumerary kidneys: A very rare case report and review of literature. Int Med Case Rep J. 2022;15:61–68. 10.2147/imcrj.s352605 35221730 PMC8880725

[8] Mesfin T, Haji N, Seyoume F, et al. Supernumerary kidneys associated with disorders of sexual development and cloacal anomaly: A case report. Int Med Case Rep J. 2023;16:193–199. 10.2147/imcrj.s403690 36994442 PMC10042168

[9] Keskin S, Batur A, Keskin Z, Koc A, Firat Ozcan I. Bilateral supernumerary kidney: A very rare presentation. Iran J Radiol. 2014;11(4):e11069. 10.5812/iranjradiol.11069 25780543 PMC4347730

[10] Kumar M, Kumar G, Barwal K, Raina P. Right supernumerary kidney: A rare entity. Urol Case Rep. 2019;23:97–98. 10.1016/j.euacr.2019.01.001 30729095 PMC6352298

[11] Rehder P, Rehwald R, Böhm JM, et al. Supernumerary kidneys: A clinical and radiological analysis of nine cases. BMC Urol. 2019;19(1):93. 10.1186/s12894-019-0522-0 31623590 PMC6798430

